# Serial Mediation Model of Social Capital Effects over Academic Stress in University Students

**DOI:** 10.3390/ejihpe12110115

**Published:** 2022-11-16

**Authors:** Mario Eduardo Castro Torres, Pablo Marcelo Vargas-Piérola, Carlos F. Pinto, Rubén Alvarado

**Affiliations:** 1 Universidad Mayor Real y Pontificia de San Francisco Xavier de Chuquisaca, Casilla, Sucre 212, Bolivia; 2 Independent Researcher, Calle Ladislao Cabrera, Sucre 482, Bolivia; 3 Department of Public Health, School of Medicine, Faculty of Medicine, University of Valparaíso, Valparaíso 89000000, Chile

**Keywords:** social capital, social support, self-efficacy, self-esteem, academic stress

## Abstract

Background: Although several studies have shown that social capital and social support decreases academic stress (AS), there has been lack of atheoretical model to explain how this occurs. This study aims to verify a model that explains the effect of bonding social capital (BSC) over academic stress psychological symptoms (PsyS), considering the multiple sequential mediation of socio-emotional support (SES), self-efficacy (sEffic) and self-esteem (sEstee). Methods: In a transversal study, 150 undergraduate volunteer students were recruited using non-probabilistic purposive sampling. Data were collected using psychological questionnaires and were processed through partial least squares structural equation modeling (PLS-SEM). Results: Goodness of fit of the models (SRMR = 0.056, 0.057, <HI95) (dULS, dG < HI95), reliability and validity are adequate. The indirect effect of BSC over PsyS (β = −0.196; IC 95% [−0.297, −0.098]) is relevant and significant and is serial mediated by SES and sEffic. Conclusions: From a very precise conceptual definition, a model is generated, within which empirical evidence explains the relationship between BSC and PsyS, emphasizing the role of BSC in the development of personal resources to cope with AS. This can be applied to policies and public health programs that affect these variables.

## 1. Introduction

Stress, as well as its associated pathologies (depression and anxiety), occur in various social contexts and institutions, such as the educational field. In addition, several studies have demonstrated the existence of academic stress (AS), which usually increases as higher levels of studies are reached, and higher levels universities have the highest levels of AS [1,2].

Empirical studies have shown that social capital [3,4], social support [5,6], self-esteem (sEstee) and self-efficacy (sEffic) [7,8] reduce the indicators (or effects) of general stress on university students. Studies on AS conducted in Latin America focused on their correlation with psychological variables—social support, anxiety, depression, suicidal ideation, coping styles, sEffic and sEstee—as well as sociodemographic—sex, socioeconomic level and type of family—[9,10]. However, there are no specific studies on the relationship between social capital and AS. Outside of Latin America, Changmin Yoo [11,12] found that bonding social capital (BSC) decreases indicators of AS in university and secondary school students.

While several studies have shown that both social capital and social support positively influence mental health, as well as reduce stress levels, thus far no theoretical model has been devised that can explain how this relationship occurs because of plurality in the definitions of these constructs and even due to inaccuracies in their conceptualization [13,14].

This paper aims to generate a model that explains the effect of social capital on AS. To this end, initially, the involved constructs are precisely defined, then, an explanatory model is proposed.

### 1.1. Literature Review

From the cognitive model, AS is a systemic process by which demands—internal and/or external—of the university environment are evaluated by the students as a threat to their integrity—biological and psychological—and to the quality of their meaningful social relationships when they perceive that they do not possess the necessary resources—external and/or internal—to meet the demands; in other words, stress only occurs when demands exceed their coping capability. External demands correspond to expectations, requests or requirements of significant people, as well as to social demands or limitations; internal demands are personal expectations based on one’s own values, goals, and beliefs. On the other hand, external resources are provided by the social environment and are both tangible, i.e., material, and intangible, i.e., socioemotional; finally, internal resources correspond to personal characteristics, i.e., emotional self-regulation, knowledge, problem-solving ability, sEffic and sEstee) [15,16,17].

The perception of threat produces a systemic imbalance between the student and his environment due to the possible loss of resources that allow survival, either directly or indirectly [18,19]. Imbalance manifests itself in symptoms of AS: physical, psychological and behavioral. Physical symptoms include sleep disturbances, chronic fatigue, headaches, muscle aches and digestive problems. Psychological symptoms (PsyS) include cognitive symptoms, such as problems with concentration, attention, memory and reasoning, as well as emotional symptoms, such as impatience, impulsiveness, the inability to relax, irritability and sadness. Behavioral symptoms include changes in food intake, increased frequency of personal conflicts and isolation [15,20,21].

Symptoms are the most objective indicators for assessing stress levels. In addition, they are the best predictors of the consequences of stress on mental and physical health, both in the medium- and long-term [22,23,24]. As far as AS is concerned, PsyS (cognitive as well as emotional) are the most common and have a greater relationship with stressors [25,26].

In the presence of symptoms, students develop coping strategies to regain balance; coping is defined as “a dynamic process, cognitive and behavioral, aimed at activating the necessary resources to meet the demands” [15] (p. 20). These resources, both internal and external, are related to social networks, social capital and social support.

From a structural relational approach, the social network is a structure made up of a defined set—i.e., with clear limits—of individuals or groups connected to each other by multiple ties or relationships; it is a product of continuous transactional interactions of tangible and intangible resources [27,28,29]. Social networks are the basis of social capital [30,31].

Social capital is the sum of resources, potential and real, tangible—e.g., money, titles, real state—and intangible—e.g., education, social status, political power—accumulated by a person through their access to a network of stable, reliable and reciprocal social connections. In other words, social capital includes: (a) the embedded available resources, derived from the exchange process that takes place in the social network. (b) The social network itself, which is considered to be an additional resource. These resources are used to meet objectives, meet needs or to cope better with unfavorable situations [32,33].

From the configuration, characteristics and functions of the connections of social networks and social capital can be divided into dimensions. The most widely used in research are: (a) BSC and (b) Bridging social capital [30,34,35]. BSC has a bigger effect on AS [11] and on general stress [36] in university students, reducing stress symptoms.

Several studies demonstrate that psychological symptoms of stress are associated with a low level of social capital. Thus, a higher prevalence of these symptoms was found in Chinese university students with reduced social capital (i.e., low level of economic and social resources) [37]. In addition, a higher level of the same symptoms (i.e., depression, anxiety) was found in Chinese medical students with low level of social capital (i.e., living alone, with economic difficulties, low access to social support and with a dysfunctional family network) [38]. Finally, these symptoms (i.e., depression, anxiety) are higher in low-income high school students in Norway [39].

The process by which accumulated resources (i.e., social capital) are employed to meet needs is called social support. More precisely, social support is “the process (e.g., perception or reception) by which the resources of the social structure are used to meet functional (e.g., instrumental and expressive) needs in routine and crisis situations” [40] (p. 383). This process can be tangible (i.e., actual receipt of resources) or only perceived (i.e., to assume that such resources are available or may be received thereafter) [41]. BSC is the main source of social support [41,42,43,44], as it is based on strong social ties or relationships, where contact is frequent, trust and reciprocity are high, and more intense emotional attachments are held [31,45,46].

Depending on the needs covered by social support, it can be divided into: (a) Socio-emotional support (SES), which is affective as well as expressive-informative and includes the reception and/or perception of being able to receive affirmations and demonstrations of love, value and acceptance, as well as listening, advice, guidance and feedback. (b) Instrumental support, which is the reception and/or perception of receiving material resources or services [41,42,43,44].

SES is shown to have more effects on AS [12] and general stress [36,47] in university students, reducing symptoms or indicators of stress. This happens because social resources—i.e., social capital—are channeled through SES to meet expressive and emotional needs, generating two internal—i.e., psychological—resources, basic for stress coping: sEffic and sEstee [48,49,50,51].

Perceived sEffic is shaped by a person’s beliefs regarding the ability to regulate his/her own performance and be able to control events affecting their lives [52,53]. Furthermore, “efficacy beliefs influence how people think, feel, motivate and act” [54] (p. 2). On the other hand, sEstee is the “positive or negative attitude toward the self as a totality” [55] (p. 141). It includes two components: (a) Cognitive, that is, beliefs about oneself, and (b) Affective, the feelings that these beliefs generate. In addition, the affective component determines the intensity and its positive or negative character. In addition, sEstee is the result of a self-assessment primarily linked to sEffic and social acceptance; that is, sEstee depends on the degree to which a person is considered accepted on their social networks for possessing attributes that are valued on the same network [44,55,56]. For this reason, sEffic increases the levels of sEstee [57,58]. In university students, sEffic reduces levels of AS [59] and general stress [60,61] while sEstee only reduces the latter [47,61,62].

### 1.2. Research Model and Hypothesis

**Figure 1:**
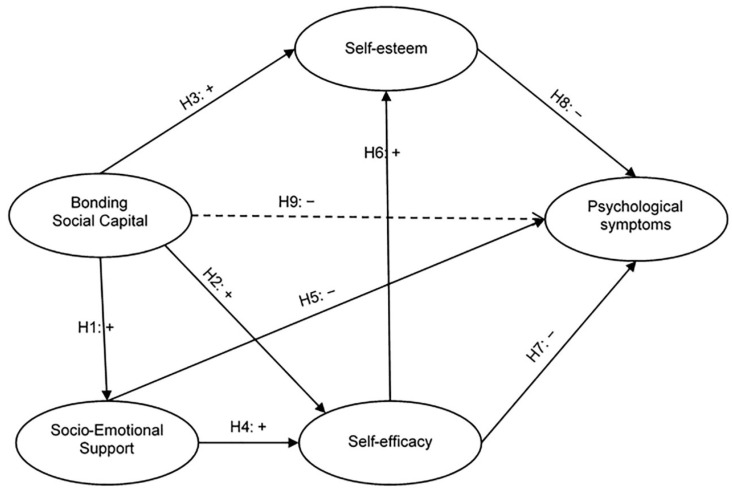
Hypothesis model.

Based on the conceptual review presented, it is considered that the BSC, through the SES, provides both social (external) and psychological (internal) resources that influence the AS process of university students. More specifically, it is proposed that the effect of BSC on PsyS is mediated by SES, sEffic and sEstee. The model is presented in Figure 1:

Thus, the following hypotheses arise:

**H1:**  *BSC has a positive and direct influence on SES levels*.

**H2:**  *BSC has a positive and direct influence on levels of sEffic*.

**H3:**  *BSC has a positive and direct influence on sEstee levels*.

**H4:**  *SES has a positive and direct influence on levels of sEffic*.

**H5:**  *The SES has a negative and direct influence on the PsyS*.

**H6:**  *sEffic has a positive and direct influence on levels of sEstee*.

**H7:**  *sEffic has a negative and direct influence on PsyS*.

**H8:**  *sEstee has a negative and direct influence on PsyS*.

**H9:**  *BSC has an indirect negative effect on PsyS*.

## 2. Materials and Methods

### 2.1. Research Design and Setting

The study uses a cross-sectional design, based on questionnaires, to investigate the relationship between BSC and PsyS of AS—with serial mediation of SES, sEffic and sEstee—in university students.

### 2.2. Sample Size

The sampling by convenience method was used to obtain the sample from this study. The minimum sample size was calculated with statistical software G*Power, version 3.1.9.6 [63] was 85 (1 − β = 0.8; α = 0.05; f2 = 0.15) [64,65]. However, 150 volunteer university students from the Faculty of Humanities of the Major, Royal and Pontifical Saint Francis Xavier of Chuquisaca University [Universidad Mayor Real y Pontificia de San Francisco Xavier de Chuquisaca] participated in Sucre, Bolivia. Nevertheless, 25 were excluded due to incomplete answers, with 125 people remaining in this study; their ages ranged between 18 and 21, with an average of 19 years; 102 (81.60%) were women and 23 (18.40%) men.

### 2.3. Instruments

To measure the BSC, the Personal Social Capital Scale (PSCS-16) was used, with 16 items on a Likert scale, and with five response alternatives. Its original reliability is adequate (α = 0.90) and has optimal criterion-related and internal validity [66].

To evaluate sEstee, the Rosenberg Self-Esteem Scale (RSES), which has 10 items on a Likert scale with four response alternatives, was used [55]. A version translated to Spanish and validated in university students, with adequate reliability (α = 0.88) and optimal criterion-related validity, was employed [67].

To measure PsyS, the Academic Stress Inventory (SISCO) was used, which assesses the intensity and four dimensions of the AS. This study used only the Psychological Symptoms dimension, with 5 items on a Likert scale and with five response alternatives. The original scale has good reliability (α = 0.90) and optimal validity based in the internal structure [68].

The Medical Outcomes Study Social Support Scale (MOS-SSS) questionnaire, from Sherbourne and Stewart, which evaluates three dimensions of social support, was used to measure SES [69]. In this research, the Emotional Support and Affective Support dimensions of a version translated and validated in Spain were applied, with 13 items on a Likert-type scale and with five response alternatives; it has very good reliability (α = 0.941) and optimal criterion and internal structure-based validity [70].

To estimate sEffic, Perceived Stress Scale (PSS-14), by Cohen et al., was employed [71]. A version translated and validated in Spain, with adequate reliability (α = 0.81) and optimal criterion validity, was used [72], with seven items on a Likert-type scale and with five response alternatives corresponding specifically to sEffic [73,74].

### 2.4. Procedures

The research profile was approved by Bolivia’s National Bioethics Committee. Subsequently, authorization was obtained from the University authorities and the voluntary participation of the students was requested. Those who agreed to participate collectively responded to the battery of tests in university classrooms between November 11 and 15, 2019.

### 2.5. Statistical Data Analysis

Partial least squares structural equations modeling (PLS-SEM) was employed as it allows: predicting the behavior of the variables of the model proposed based on the theoretical review; understanding a complex phenomenon, which includes several constructs and mediation relationships; and the analysis of complex models with small samples, without requiring a normal distribution of the data [64]. The SmartPLS program (Version 3.3.9) [75] was used with the following parameters: (1) Estimation method: Path weighting scheme; (2) Data metric: Raw data; (3) Initial values to start the PLS-SEM algorithm: +1; (4) Stop criterion: 1 × 10^−7^ (0.0000001); (5) Maximum number of iterations: 300 [65].

To test the model, the two-stage disjoint approach for higher-order constructs [76] was used, as the BSC variable is precisely a higher-order construct, consisting of four dimensions, each corresponding to a lower-order construct: network size, trust in network members, ownership of resources and reciprocity [66].

In order to empirically test the theoretical model proposed, the goodness of model fit of the measurement and structural models were evaluated using the saturated and estimated models, respectively, employing: (a) the value of the standardized residual mean square root (SRMR), (b) the distance of unweighted least squares (d_ULS_), and (c) the geodesic distance (d_G_); to have an adequate adjustment level, values of SRMR, d_ULS_ and d_G_ must be less than the 95% quantile (HI_95_) of their reference distribution. In addition, the SRMR value must be < 0.08. To assess the validity and reliability of the measurements of the constructs and their components, both of the first and second order, we considered: (1) indicators reliability by factorial loading (λ ≥ 0.708); (2) construct reliability by means of the Dijkstra-Henseler Rho index (ρA ≥ 0.707); (3) convergent validity by the mean extracted variance (AVE > 0.5); and (4) discriminating validity by means of the proportion of heterotrait-monotrait correlations (HTMT < 0.85 or HTMT < 0.90, 95% CI ≠ 1). To test the hypotheses, the relations among constructs of the structural model were evaluated. To determine the direct effects: (A) the algebraic sign of the route coefficients (β) was equal to the one raised in the hypotheses; (B) the magnitude or level of the relationship between the constructs (relevance) (β > 0.1); and (C) the statistical significance (importance) of the values of (β) through bias-corrected and accelerated bootstrap confidence intervals (BCa), with a significant confidence level of 5% that should not include zero (95% ≠ 0 CI), obtained with a bootstrap of 5000 samples. For the evaluation of total indirect effects, the same parameters were considered, and for the evaluation of partial indirect effects, only the algebraic sign and significance. Finally, the predictive power within the sample was evaluated by the coefficient of determination (R^2^ > 0.1) and the predictive power outside the sample with the Stone-Geisser Index (Q^2^ > 0) was obtained by the blindfolding procedure, under the cross-validation redundancy approach [64,65,77,78,79,80].

## 3. Results

**Figure 2:**
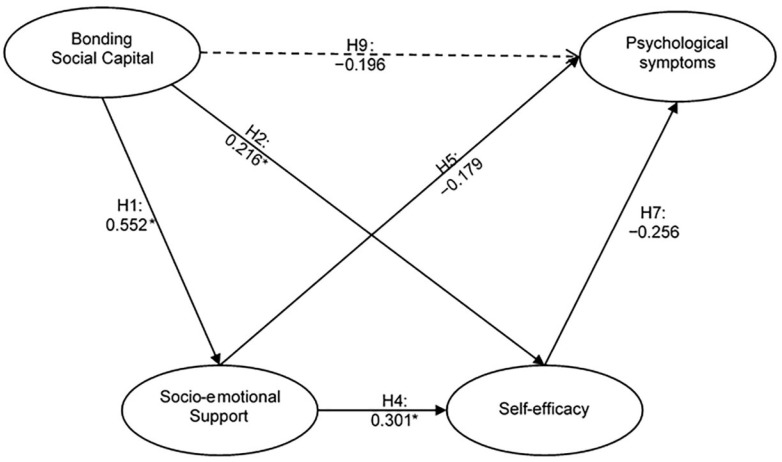
Empirical model: Results of the structural model. Note: Segmented lines represent indirect relations. * CI 95% ≠ 0.

According to the initial results of the model evaluation, the indicators with factorial loadings < 0.708, and/or determining that the AVE > 0.5, were removed, as well as the variable sEstee. Subsequently, the final analysis of the model was carried out, the results of which are presented in Figure 2.

### 3.1. Assessment of Overall Fit of Measurement and Structural Models

The results of the goodness of model fit of the measurement and structural models are presented in Table 1. In both models, the SRMR < 0.08 and d_ULS_ and d_G_ are less than the 95% (HI_95_) quantile of their reference distribution, demonstrating an acceptable adjustment of the measurement model and showing that there is empirical evidence to support the estimated model with a 5% level of significance.

### 3.2. Measurement Model (Outer Model)

All items have a factorial load λ > 0.708, indicating that the items belong to the proposed constructs. In addition, all constructs have the Dijkstra-Henseler Rho index ρA > 0.707; therefore, the scores of latent variables correspond to the constructs. Similarly, these constructs have an average variance extracted AVE > 0.50, which suggests that the indicators measure the constructs to which they belong (unidimensionality) and, therefore, have convergent validity. This can be seen in Table 2.

Finally, all of the values of the heterotrait-monotrait (HTMT) ratio of correlations are below the recommended threshold and the 95% confidence interval does not include one (1), which demonstrates the discriminating validity of the constructs: there is a statistically significant difference among the latent variables of the model. These results can be seen in Table 3.

These results show that the measures of the employed instruments have acceptable characteristics of reliability and validity. Regarding reliability, the results indicate that the construct scores are free of random errors because more than 50% of the variance of the scores of the items and of the tests are explained specifically by the construct they measure (internal consistency); consequently, construct scores are assumed to be reliable. On the other hand, the results show that the measures of each latent variable statistically converge toward their own construct and diverge from others (convergent validity). Moreover, each construct is unique, as there is a statistically significant difference between the latent variables of the model, so each one represents a single theoretical concept of the model (discriminant validity).

### 3.3. Structural Model (Inner Model)

The results demonstrate that BSC has a positive and direct influence on SES (β = 0.552, CI 95% [0.411, 0.653]) (H1) and also over the levels of perceived sEffic (β = 0.216, CI 95% [0.007, 0.404]) (H2). In addition, SES has a positive and direct influence on sEffic (β = 0.301, CI 95% [0.090, 0.485]) (H4). Finally, the results confirm that SES (β = −0.179, CI 95% [−0.335, −0.003]) (H5) and sEffic (β = −0.256, CI 95% [−0.402, −0.034]) (H7) have a negative and direct influence on the levels of PsyS of AS.

In summary, the five proposed relationships coincide with the algebraic sign raised, and are shown to be relevant and statistically significant. Therefore, hypotheses 1, 2, 4, 5 and 7 were supported and accepted. On the contrary, hypotheses 3, 6 and 8 did not enter the final model and were rejected. These results are shown in Table 4

The analysis of the mediation hypotheses focused on the interpretation of the indirect effect of BSC on PsyS through two intermediate constructs: SES and sEffic. As shown in Table 4, the proposed relationship coincided with the algebraic sign raised. The results of the total and specific indirect effect confirm that the BSC has a negative and indirect effect on PsyS by three routes (β = −0.196, CI 95% [−0.297, −0.098]). The relationship is relevant (magnitude > 0.1) and both the total and specific effects are statistically significant as the confidence interval does not include zero (0); therefore, hypothesis 9 was supported and accepted.

As shown in Table 5, the value of the coefficients of determination (R^2^) of the variables > 0.1, therefore, the model has internal predictive relevance. In addition, the Stone-Geisser Index (Q^2^) > 0, so the model also has external predictive relevance.

## 4. Discussion

The objective of this study was to empirically test a model on the relationship between BSC and PsyS, where BSC provides internal psychological resources (sEffic and sEstee) through the SES, which affect the PsyS level on university students; more precisely, the effect of BSC over PsyS is mediated by SES, sEffic and sEstee.

The results show that the higher the level of BSC, the higher the perception of SES and sEffic; and these variables in turn increase the likelihood that university students will have lower levels of PsyS. In other words, the BSC indirectly, negatively, relevantly and significantly influences PsyS through multiple and serial mediation of SES and sEffic. Therefore, there is a higher likelihood that students with higher levels of BSC will have lower levels of PsyS due to SES and sEffic transmitting the effects of BSC on PsyS. This result is partially related to Kalaitzaki’s study [36], which indicates that there is a negative effect of BSC on overall stress in university students. In addition, this study relates to the works of Yoo [11], who found that BSC is a causal predictor of AS and that BSC originated in family ties has a negative effect on AS. Similarly, this study relates to the research of Chen [81], who reports that there is a negative effect of BSC on stress in the general population.

Next, the results of the proposed model are analyzed in detail. First, BSC has a direct, positive, relevant and significant effect on SES and sEffic. These findings are consistent with the results of Yoo’s work [11], who found that the BSC has a positive direct effect on social support. In addition, the results are consistent with the results of Brouwer et al. [82], who identified that social capital increases the level of sEffic in university students and, as a result, increases their success in their studies. In addition, these results are similar to the work of Liu and Ngai [50], which shows that social capital favors the development of a healthy identity in adolescents through the mediation of sEffic. Finally, they are consistent with the study of Ramos et al. [83] who found a direct positive effect of social capital on sEffic. Moreover, SES has a direct, positive, relevant and significant influence on sEffic. This result is similar to the findings of Bayani [48] and Ozer [51], who found the same effect in students. Therefore, students with a higher levels of SES and sEffic are likely to have a lower level of PsyS.

Finally, the results show that SES and sEffic directly, negatively, relevantly and significantly influence PsyS. These findings coincide with Ozer’s work [51], which identifies that SES and sEstee decrease the level of perceived stress in university students. They are also similar to Bayani’s research [48], which shows that SES and sEffic decrease the level of AS in reference to examinations. Similarly, it is consistent with the study of Abid et al. [59], which identified that in university students there is a negative correlation between sEffic and AS. Therefore, students with a higher level of SES are likely to have a lower level of PsyS.

The hypotheses related to the sEstee variable were rejected because it was not taken into account in the final model, as it did not present relationships with the adequate level of relevance and significance.

The model developed in this research corroborates several theories, but primarily the approach of Thoits [43,44], who proposed that: (i) Belonging to social networks is the foundation of effectiveness, based on trust and reciprocity; (ii) Social support is a process and emotional support is the most important for health outcomes; (iii) sEffic is a result of social support; (iv) sEffic plays an important role in coping with stress.

As with any study, this work also presents limitations. First, the characteristics and size of the sample are not representative of all university students in Bolivia. Secondly, as it is a cross-sectional study, it does not allow for establishing conclusive causal relations. Despite these limitations, the study presents empirical evidence supporting the explanation of the relationship that was analyzed.

Therefore, it is recommended to check the model presented with larger and more representative samples, as well as the development of cohort studies to verify the directionality of the associations. In addition, it is suggested to expand the model by including other variables, such as personality, specific stressors and the relationship among PsyS and mental health aspects, especially depression.

## 5. Conclusions

The findings of this study confirm the results of other empirical works that, separately, identified the impact of BSC, SES and sEffic on AS. However, this study explains it from a model that integrates all these variables, which constitutes its main contribution. While this is an important step towards understanding a complex phenomenon, the model verified in this study must be replicated and expanded, as the effects of other variables still need to be identified, given the complex characteristics of any social phenomenon.

Moreover, this study conceptually defines the variables social capital and social support in a clear and concise way, which is of great important because the confusion between these concepts did not allow a full understanding of their effect on stress. Therefore, an explanatory model could not previously be proposed, and is achieved in this study. 

This study demonstrates the outstanding role of social capital in the proper management of stressful events, as it allows the development of other internal resources, such as sEffic, which are not only limited to enabling adequate stress management, but are also fundamental for dignified personal development and to improve the quality of life.

Given the complex relationships identified in this study, it would be relevant to include social capital and social support in social intervention work that seeks to improve the level of sEffic and/or decrease the level of AS in university students. This way, universities should develop public health policies and programmes in this regard to improve the quality of life and academic performance of their students.

## Figures and Tables

**Table 1:** Overall models fit evaluation.

Discrepancy	Value	HI_95_	Conclusions
Saturated Model			
SRMR	0.056	0.061	Supported
d_ULS_	0.869	1.013	Supported
d_G_	0.528	0.640	Supported
Estimated Model			
SRMR	0.057	0.064	Supported
d_ULS_	0.891	1.042	Supported
d_G_	0.529	0.640	Supported

**Table 2:** Evaluation of the measurement model.

Construct	Indicator	λ ^a^	ρA ^b^	AVE ^c^
First order constructs				
BSC-Tr			0.882 ***	0.876 ***
	Item 3	0.948 ***		
	Item 4	0.924 ***		
BSC-RP			0.737 ***	0.788 ***
	Item 5	0.875 ***		
	Item 6	0.900 ***		
BSC-Re			0.919 ***	0.920 ***
	Item 7	0.955 ***		
	Item 8	0.963 ***		
BSC-NS			0.776 ***	0.813 ***
	Item 1	0.912 ***		
	Item 2	0.890 ***		
Socio-emotional support			0.958 ***	0.700 ***
	SES-3AE	0.851 ***		
	SES-4AE	0.838 ***		
	SES-8AE	0.801 ***		
	SES-9AE	0.855 ***		
	SES-13AE	0.867 ***		
	SES-16AE	0.865 ***		
	SES-17AE	0.888 ***		
	SES-19AE	0.850 ***		
	SES-6AA	0.811 ***		
	SES-10AA	0.802 ***		
	SES-20AA	0.770 ***		
Self-efficacy			0.821 ***	0.624 ***
	sEffic-6	0.723 ***		
	sEffic-7	0.841 ***		
	sEffic-9	0.850 ***		
	sEffic-10	0.739 ***		
Psychological Symptoms			0.882 ***	0.698 ***
	PsyS-18	0.754 ***		
	PsyS-19	0.864 ***		
	PsyS-20	0.890 ***		
	PsyS-21	0.828 ***		
Second-order constructs				
Bonding social capital			0.891 ***	0.749 ***
	BSC-Tr	0.881 ***		
	BSC-RP	0.836 ***		
	BSC-Re	0.898 ***		
	BSC-NS	0.846 ***		

^a^ Factorial loadings, ^b^ Dijkstra-Henseler Rho, ^c^ Average variance extracted. *** *p* ≤ 0.001, based on *t* test (4999) of one tailed. Note. BSC-Tr = Bonding Social Capital—Dimension Trust; BSC-RP = Bonding Social Capital—Dimension Resources Property; BSC-Re = Bonding Social Capital—Dimension Reciprocity; BSC-NS = Bonding Social Capital—Dimension Net Size.

**Table 3:** Evaluation of measurement model: discriminant validity (HTMT).

Construct	1	2	3	4	5	6	7	8
First order constructs								
(1) Socio-emotional support								
(2) Self-efficacy	0.479 *							
(3) BSC-Tr	0.530 *	0.393 *						
(4) BSC-RP	0.505 *	0.431 *	0.828 *					
(5) BSC-Re	0.585 *	0.350 *	0.827 *	0.836 *				
(6) BSC-NS	0.520 *	0.449 *	0.783 *	0.794 *	0.840 *			
(7) Psychological Symptoms	0.312 *	0.373 *	0.171 *	0.207 *	0.111 *	0.201 *	-	
Second-order constructs								
(8) Bonding social capital	0.596 *	0.443 *	-	-	-	-	0.182 *	-

* CI 95% ≠ 1.

**Table 4:** Structural model assessment.

Hypothesis	Relation	(β)	IC 95% (^a^)		Conclusions
			LL	UL	
Direct effects					
H1 (+)	BSC → SES	0.552	0.411	0.653	Supported
H2 (+)	BSC → sEffic	0.216	0.007	0.404	Supported
H4 (+)	SES → sEffic	0.301	0.090	0.485	Supported
H5 (−)	SES → PsyS	−0.179	−0.335	−0.003	Supported
H7 (−)	sEffic → PsyS	−0.256	−0.402	−0.034	Supported
Total indirect effect					
H9 (−)	BSC → PsyS	−0.196	−0.297	−0.098	Supported
Specific indirect effects					
	BSC → SES → PsyS	−0.099	−0.195	−0.002	Supported
	BSC → sEffic → PsyS	−0.055	−0.124	−0.003	Supported
	BSC → SES → sEffic → PsyS	−0.043	−0.106	−0.007	Supported

Note. CI = confidence interval; LL = lower limit; UL = upper limit; BSC = bonding social capital; SES = socio-emotional support; sEffic = self-efficacy; PsyS = psychological symptoms of AS. ^a^ Bias-corrected and accelerated (BCa) bootstrap confidence intervals for 5% probability of error (α = 0.05).

**Table 5:** Evaluation of internal (R²) and external (Q²) predictive relevance.

Construct	R²	Q²
PsyS	0.122	0.086
SES	0.299	0.203
sEffic	0.196	0.119

Note. SES, socio-emotional support; SEffic, self-efficacy; PsyS, psychological symptoms of academic stress.

## Data Availability

The data presented in this study are available on request from the corresponding author. The data are not publicly available due to privacy and confidentiality.
